# Within- and across-day patterns of interplay between depressive symptoms and related psychopathological processes: a dynamic network approach during the COVID-19 pandemic

**DOI:** 10.1186/s12916-021-02179-y

**Published:** 2021-11-30

**Authors:** Omid V. Ebrahimi, Julian Burger, Asle Hoffart, Sverre Urnes Johnson

**Affiliations:** 1 Department of Psychology, University of Oslo, Oslo, Norway; 2 Modum Bad Psychiatric Hospital and Research Center, Vikersund, Norway; 3 Department of Psychology, University of Amsterdam, Amsterdam, The Netherlands; 4 Centre for Urban Mental Health, University of Amsterdam, Amsterdam, The Netherlands; 5 University Medical Center, University of Groningen, Groningen, The Netherlands

**Keywords:** Dynamic network analysis, Depression, Psychopathological mechanisms, Longitudinal study, Nomothetic time series analysis, General adult population, COVID-19 pandemic

## Abstract

**Background:**

In order to understand the intricate patterns of interplay connected to the formation and maintenance of depressive symptomatology, repeated measures investigations focusing on within-person relationships between psychopathological mechanisms and depressive components are required.

**Methods:**

This large-scale preregistered intensive longitudinal study conducted 68,240 observations of 1706 individuals in the general adult population across a 40-day period during the COVID-19 pandemic to identify the detrimental processes involved in depressive states. Daily responses were modeled using multi-level dynamic network analysis to investigate the temporal associations across days, in addition to contemporaneous relationships between depressive components within a daily window.

**Results:**

Among the investigated psychopathological mechanisms, helplessness predicted the strongest across-day influence on depressive symptoms, while emotion regulation difficulties displayed more proximal interactions with symptomatology. Helplessness was further involved in the amplification of other theorized psychopathological mechanisms including rumination, the latter of which to a greater extent was susceptible toward being influenced rather than temporally influencing other components of depressive states. Distinctive symptoms of depression behaved differently, with depressed mood and anhedonia most prone to being impacted, while lethargy and worthlessness were more strongly associated with outgoing activity in the network.

**Conclusions:**

The main mechanism predicting the amplifications of detrimental symptomatology was helplessness. Lethargy and worthlessness revealed greater within-person carry-over effects across days, providing preliminary indications that these symptoms may be more strongly associated with pushing individuals toward prolonged depressive state experiences. The psychopathological processes of rumination, helplessness, and emotion regulation only exhibited interactions with the depressed mood and worthlessness component of depression, being unrelated to lethargy and anhedonia. The findings have implications for the impediment of depressive symptomatology during and beyond the pandemic period. They further outline the gaps in the literature concerning the identification of psychopathological processes intertwined with lethargy and anhedonia on the within-person level.

**Supplementary Information:**

The online version contains supplementary material available at (10.1186/s12916-021-02179-y).

## Background

The global pandemic caused by the SARS-CoV-2 virus has been accompanied by substantial augmentations in psychiatric symptoms in the general population, with scholars denoting this homologous co-occurrence as a parallel pandemic of detrimental psychiatric symptomatology [1]. Among the studied symptom domains, the cross-continental elevations in depressive symptoms have been deemed an area of concern warranting further investigation [2–5]. To date, the preponderance of the pandemic literature has concerted its efforts toward the identification of prevalence estimates and demographic risk factors accompanied by the alterations in depressive symptom levels [3, 6, 7]. Consequently, knowledge remains exiguous concerning the psychopathological mechanisms that are interconnected with psychiatric symptom expressions during the pandemic [7].

Psychopathological mechanisms refer to processes which contribute to the amplification and maintenance of psychiatric symptomatology. Within the processes encapsulated in this phenomenon, behavioral and cognitive-affective mechanisms connote a prime category of interest, as they are loanable to manipulation by a wide range of psychiatric treatment modalities aimed at alleviating depression. Notably, such mechanisms (e.g., rumination) entail processes that are tied to fluctuations in symptoms *within* individuals. By contrast, risk factors provide information about the likelihood of experiencing detrimental symptoms *compared to peers* in the population with other dispositional or circumstantial disparities. Accordingly, investigations of mechanistic processes versus risk factors of depression yield distinctive pieces of information not necessarily compatible with the other, with their separation requiring the deployment of the appropriate level of analysis to disaggregate between what is referred to as *within-person* and *between-person* relationships, respectively [8]. As reflected by recent research calls [7, 9], however, much of the pandemic literature encompasses of study designs and analytical tools that are precluded from appropriate separation of these pivotal relationships.

Several scholars have denoted the substantive necessity of disentangling within- from between-person relationships [8, 10–14], with an example from the field of medicine highlighting its importance. Although the risk of heart attack is lower among physically active people (i.e., a *between-person* relationship), the chances of an individual having a heart attack is higher while exercising (i.e., a *within-person* relationship). Consequently, the presence of these opposing effects with the same set of variables (termed Simpson’s paradox, e.g., [14]) accentuates the importance of their appropriate and distinctive investigation. From this perspective, knowledge concerning the formation of depressive symptoms and their patterns of interconnection with psychopathological mechanisms warrants investigations at the within-person level of analysis, presenting a key step toward the identification and impediment of the escalatory processes tied to the aforementioned increases in detrimental depressive symptomatology during the present pandemic. Mapping out such interrelations is further of utility beyond the pandemic period, as more knowledge is needed concerning the multitudinous processes involved in the maintenance of deleterious mental health states in non-clinical populations. As such, calls have been made for the adaptation of multi-level dynamic network approaches using longitudinal designs and time series data [4, 7, 15–17], yielded with the aptitude of detecting the different components involved in the maintenance of depressive symptomatology while appropriately separating within- from between-person effects across time.

A suitable dynamic network approach incorporating these properties includes the use of the multi-level vector autoregressive (VAR) model, further allowing investigations of relationships among variables occurring across specific time lags and within a given time window [18–20]. These patterns of interaction may further be interpreted through the lens of the network theory of mental disorders [21, 22], conceptualizing psychiatric symptoms and related components as networks of causally interacting entities. The time-lagged relationships in such dynamic network models are indicative of *Granger causal* relationships [23], denoting a variable’s ability to predict another variable at the consecutive time point, yielding important information about which variable temporally precedes another in a system. Simultaneously, such network models provide information concerning interactions between variables occurring within a given time window, providing information about processes that may unfold at a faster rate than the studied temporal window of measurement [24]. In summary, the adaptation of dynamic network models allows for investigations of within-person relationships between symptoms and mechanisms, while providing information about their temporal order and preliminary indications concerning the time windows which they interact on.

The present preregistered study uses multi-level VAR networks to investigate the day-to-day and within-day fluctuations of depressive symptoms during the COVID-19 pandemic, with the aim of identifying the mechanistic processes involved in the amplification and maintenance of deleterious depressive symptomatology in the general adult population. In adapting a multi-level approach, the study further disentangles within-person from between-person relationships to identify and separate between processes of change and risk factors, respectively. Such investigations represent tests of theorized connections between depressive symptomatology and its constituents, advancing the insight concerning the patterns of interplay present among symptoms and mechanistic and contextual variables in detrimental depressive states.

As detailed in the preregistered protocol of this study, a comprehensive range of psychopathological mechanisms and contextual variables were investigated, with the aim of advancing the insight concerning how these theorized variables interact with specific symptoms of depression. Several psychopathological theories predict rumination to be a key process involved in depressive dynamics. As proposed by metacognitive theory [25], rumination may arise as an attempt to understand the reasons of depressed mood, only to operate as a maintaining mechanism of depressive symptomatology with individuals remaining stuck in the depressed state through engagement in repetitive cognitive processes rather than functional problem solving. Among other psychopathological mechanisms, helplessness may play a particularly prominent role in maintaining depressive states during pandemic periods, with learned helplessness theory predicting depressive symptomatology to arise when individuals perceive to have limited influence over the circumstances they are exposed to [26]. Additionally, emotion regulation difficulties are theorized as a maintaining mechanism in depressive states, with increased proneness of employing maladaptive emotion regulation strategies presenting greater difficulties of recovering from negative emotions, sustaining the depressed mood [27, 28]. Finally, contextual variables previously tied to depressive states in prepandemic periods were investigated, including loneliness [29], physical activity [30], social media use [31], interpersonal conflict [32], sleep quality [33], relatedness needs [34], and productivity [35]. As the preponderance of these aforementioned variables has been subject to fluctuation during the present pandemic, an investigation of their relevance in the maintenance of depressive states is important. Examples include fluctuation in loneliness levels tied to social distancing protocols [36], changes in productivity related to transitions from work to home office, and sleep disturbances connected to perturbations in daily routine [37]. Finally, access to information [4] and social contact [38] was investigated, both of which have been related to depressive symptoms in pandemic settings.

## Methods

The preregistered protocol of this study can be found at the online repository of the Center for Open Science (https://osf.io/trf2y). All elements of the submitted study adhere to its preregistered protocol. Ethical approval for this study was granted by the Regional Committee for Medical and Health Research Ethics (reference: 125510).

### Study design and time period description

The present study comprises an intensive longitudinal design conducting daily measures of depressive symptomatology and related mechanistic and contextual constituents for 40 consecutive days during the COVID-19 pandemic. This data collection method is referred to as a diary study and falls under the area of ambulatory assessment techniques [39], which also encompass the experience sampling method (ESM) and ecological momentary assessment (EMA). In the clinical empirical literature, these terms are often used interchangeably and commonly referred to as the sampling of intensive longitudinal data in the participant’s real life using portable devices.

The measurement period (i.e., February 17 to March 28, 2021) was characterized by several periodic-specific events, encompassing (a) three longer and continuous periods of national holidays (i.e., days 6 to 12, days 13 to 19, and day 38 onward) and (b) a consecutive and uninterrupted period with implemented viral mitigation protocols where no modifications in national protocols occurred (i.e., days 20 to 37). This uninterrupted viral mitigation period was characterized by a stable set of protocols, such as quarantine upon contact with infected individuals, isolation upon infection, closure of schools and universities, restriction on social gatherings, public activities and events, and visitation restrictions. Several of these implemented protocols (e.g., social gatherings, domestic travel, and visitation restrictions) were slightly lightened during the three holiday intervals encompassed in the study period (i.e., the two winter and the Easter holidays).

### Participants and procedure

This study is part of the Norwegian COVID-19, Mental Health and Adherence Project, a large ongoing longitudinal investigation of psychiatric symptomatology in the general adult population. Eligible participants included all adults (i.e., age ≥ 18 years) residing in Norway. Prior to the aforementioned daily measurements conducted for the present study, the participants provided responses at four measurement waves since the onset of the pandemic. Upon initial recruitment to the project (i.e., the first wave of data collection, March 2020), the participants responded through an online survey disseminated to a random selection of Norwegian adults through a Facebook business algorithm, in addition to systematic dissemination of the survey via national, regional, and local information platforms (i.e., television, radio, and newspapers). This procedure is elaborated in detail elsewhere [4]. The same participants were recontacted at each wave of measurement. At the fourth wave of data collection (i.e., January 2021), the participants were queried concerning their interest in participating in an upcoming 40-day study about mental health (i.e., the present study). A total of 2383 participants expressed interest to partake in the study, of which 1706 individuals formally enrolled in the study. Daily measures were conducted across a 40-day period, encompassing of a 24-h sampling frequency with the participants receiving the set of time-variant items each evening at 18:30 (6:30 PM). The sampling frequency was held constant throughout the measurement period, and daily measures were conducted to investigate temporal effects (i.e., relationships across days) and contemporaneous effects within the same time window (i.e., relationships within a day) [24]. The daily sampling frequency was deemed as appropriate given its direct relation to the assessment of depressive symptom endorsement in the Diagnostic and Statistical Manual of Mental Disorders (DSM-V), querying about the presence of symptomatology during and across days [40].

### Measurement

#### Time-invariant variables

The participants reported their age, sex, education, civil status, preexisting mental health status, and region of residence.

#### Time-variant variables: item selection procedure and response scale

The item selection procedure in the present study was designed to accommodate for critical topics in the dynamic network analytic literature. First, all items were selected with the aim of avoiding topological overlap and thus possible inflation in centrality estimates [41]. Second, this theoretically grounded selection was proceeded by a data-driven approach, affirming the correlation matrix to be positive definite and that the included items were not linear combinations of one another. Subsequently, the goldbricker algorithm [42] was used to search for pairs of highly intercorrelated items, in addition to items displaying similar behavioral patterns with the other items in the network. Dependent correlations were investigated using the Hittner method [43]. The data analytical approach was congruous with the theoretical selection, identifying no redundant items.

Another topic that has received notable attention in the (dynamic) network literature includes utilization of validated items, which were predominantly adapted in this study (cf. preregistration protocol). Finally, these aforementioned topics were coupled with selections of theorized psychopathological mechanisms and contextual variables of potential relevance to depressive symptom dynamics. Overall, the item selection process followed a consensus procedure consisting of six meetings between the authors, yielding the following preregistered study protocol (https://osf.io/rekzm) containing the full details of each investigated variable and the theoretical rationale underlying item selection.

The full list of items measuring the depressive symptoms and related mechanistic and contextual constituents is provided in Table 1. All items were adapted to capture daily patterns of interplay. Following Fried and colleagues [44], the items were measured on a 5-point response scale, with all variables and their full measurement details presented in the table note of Table 1.

**Table 1:** All Items were measured on a 5-point scale (1–5). Items 1–13: 1 (not at all), 2 (slightly), 3 (moderately), 4 (very), and 5 (extremely). Items 14–16: 1 (0 min), 2 (1–15 min), 3 (15–60 min), 4 (1–2 h), and 5 (over 2 h). Item 17: 1 (0 min), 2 (10–15 min), 3 (15–30 min), 4 (30–60 mi), 5 (over 1 h)

No.	Abbreviation	Item
1	Depressed mood	Today, I felt down, depressed or hopeless.
2	Anhedonia	Today, I had little interest or pleasure in doing things.
3	Lethargy (energyless)	Today, I felt tired or that I had little energy.
4	Worthlessness	Today, I felt bad about myself or felt like a failure.
5	Rumination	Today, I thought negatively about things
		that have happened in the past.
6	Emotion regulation difficulties	Today, it has been difficult to cope with my emotions.
7	Helplessness	Today, I felt helpless with regard to my problems.
8	Loneliness	Today, I felt lonely.
9	Sleep satisfaction	Today, I was satisfied with my sleep.
10	Productivity	Today, I felt productive or useful.
11	Relatedness	Today, I felt close to other people.
12	Sufficient information	Today, I received enough information on how to
		deal with the pandemic and its associated protocols.
13	Interpersonal conflict	Today, I argued or had negative discussions with someone.
14	In-person social contact	Today, I spent... minutes/hours on physical social
		gatherings (i.e., meeting others face-to-face, offline).
15	Digital social contact	Today, I spent... minutes/hours on digital social gatherings.
16	Social media	Today, I spent... minutes/hours scrolling social media
		just to make the time pass.
17	Physical activity	Today, I spent... minutes/hours physically exercising to the
		extent that it lead to increased pulse or at least minimal sweating.

### Statistical analyses

#### Time series analyses and data pre-processing for network models

All statistical analyses were performed using R version 4.1.0 [45]. The R code and the correlation matrices necessary to regenerate the estimated models may be found here https://osf.io/trf2y/. Period-specific patterns across the different periods of the study (i.e., holiday periods and uninterrupted period of viral mitigation) were investigated using multilevel models, with a two-sided alpha level of.001 set as the inference criteria. Along with the time series visualizations, these auxiliary analyses provide descriptions of the investigated variables across the 40-day measurement period to be briefly presented in the “Results” section.

Prior to the estimation of the main analyses of the study (i.e., estimation of networks), pre-processing steps common for dynamic network models were performed. First, these analyses require a minimum number of observations per person. Because the procedure is based on within-person centering using sample means per person, it is generally not recommended to include individuals with less than 20 measurements [19, 46]. To find an optimal balance between including participants with minimal missingness and retaining as many participants as possible, the number of completed diaries was visualized as a function of the cumulative number of participants (see Additional file 1: Figure S1). The plot indicated that any more lenient cutoff for completed diaries than about 30 would not lead to substantially larger numbers of included participants. Accordingly, participants who completed at least 30 out of 40 diaries were selected. This resulted in including 1368 out of 1706 participants.

**Fig. 1:**
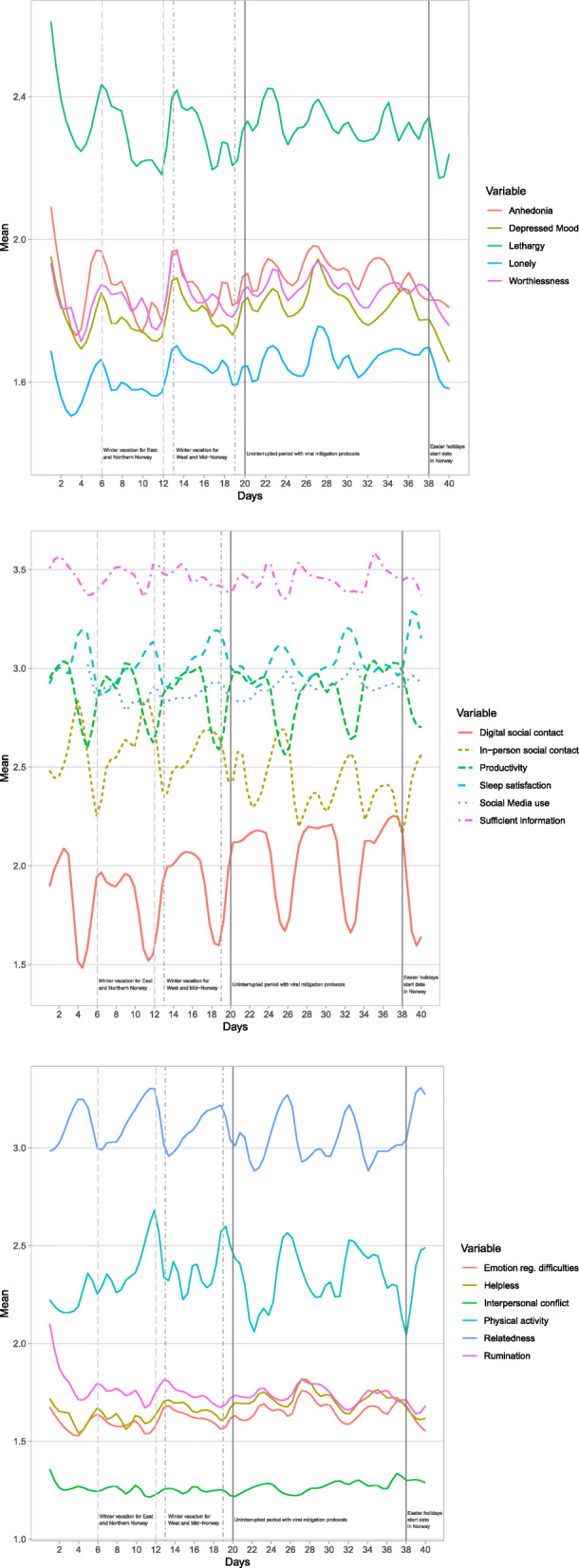
Nomothetic time series visualizations of all investigated variables through the measurement period, further depicting period-specific patterns across the 40-day study period

Second, the presence of trends in the data may lead to lower specificity or sensitivity in the resulting networks [24]. Accordingly, a linear trend analysis was performed for each variable using two components; a cumulative linear trend over the assessment period, and a weekday versus weekend trend. Such trends can be identified by performing a regression of the item scores on the assessment time (linear trend), as well as on a dummy variable coding week-days versus weekend-days (weekend trend). For a detailed, reproducible work flow of the trend removal, the reader is directed to the R code found in the “Code availability statement” section. In the subsequent analyses, these trends were removed from each variable by subtracting the linear trends and weekend effects from each observation. Note that the time series visualized in Fig. 1 portray the data prior to the detrending procedure.

#### Main analyses

We used the multi-level vector autoregressive model implemented in the *mlVAR* package in R [47] to estimate the network models from the data. The algorithm implemented in *mlVAR* is based on a two-step procedure. First, (*within-person*) temporal and *between-subjects* effects are computed based on a node-wise multi-level regression, and second, (*within-person*) contemporaneous effects are obtained by performing a subsequent node-wise multi-level regression from the residuals in step 1. In line with the recommendations for networks with more than six nodes [19, 47], *orthogonal* estimation was chosen for both the temporal and contemporaneous networks.

**Fig. 2:**
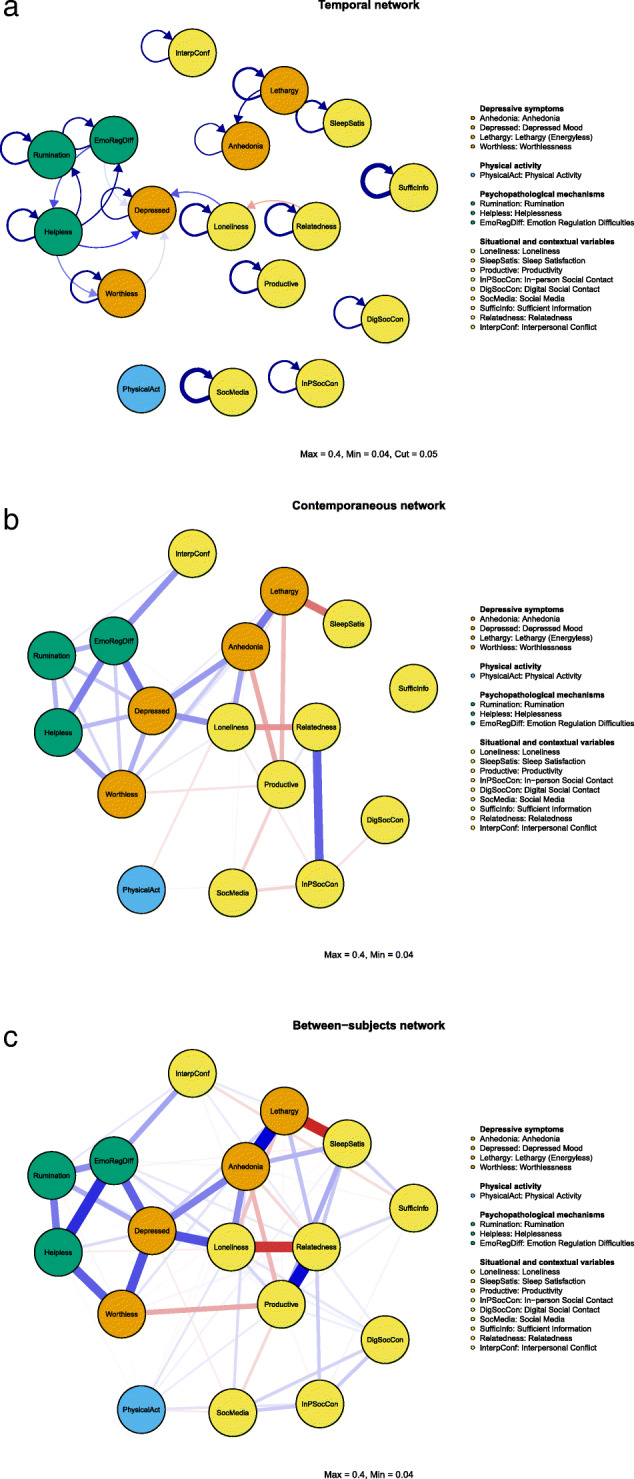
Temporal (top), contemporaneous (middle), and between-subject (bottom) networks derived from the multi-level vector autoregressive (VAR) model showing the connection between nodes while controlling for all other nodes in the network. The strongest edges include the temporal network coefficient = 0.27 (SufficInfo → SufficInfo), the contemporaneous network coefficient = 0.26 (InpSocCon ⇔ Relatedness), and the between-subject network coefficient = 0.45 (Anhedonia ⇔ Lethargy)

This results in three types of networks, visualized in Fig. 2. (1) A *fixed-effect temporal network* (top panel of Fig. 2), in which average within-person effects indicate predictions of different nodes at the consecutive time point (i.e., lag-1), capturing the potential across-day temporal interactions between depressive symptomatology and related components. The temporal network provides directed statistical relationships (i.e., one-headed arrows) that are interpreted as Granger-causal [23], representing whether a node at time *t* predicts another at the subsequent time point (i.e., *t*+1), while controlling for all other nodes in the network. (2) A *fixed-effect contemporaneous network* (middle panel of Fig. 2), indicating average within-person effects between variables that are not captured in the temporal network, which estimates the unique interactions between all nodes within the same time window. In the dynamic network literature, these effects have been interpreted as dynamics that are potentially faster than those captured in the lag-1 temporal effects [24], indicative of interactions between nodes within the same day in the present study. (3) The *between-subjects network* (bottom panel of Fig. 2) indicates relationships between variables based on the person-wise means of each variable. The between-subject network concerns average *between-person effects*, revealing how higher average levels on a variable *compared to peers* (i.e., compared to other subjects) is related to the mean levels in another variable compared to others in the population (e.g., people who on average are more physically active compared to their peers also likely have lower average heart rate than their peers). The temporal and contemporaneous networks concern average *within-person effects* across and within measured time windows respectively, both revealing how people displaying higher scores on a variable *compared to their own average* may display average within-person level changes on another variable (e.g., when individuals exert more physical activity than their own average, they also experience higher heart rate than their own average). The within-person effects provide insight into the patterns of interplay between symptoms and mechanisms of change in a depressive system, while the between-subject effects provide information concerning risk factors associated with depressive symptoms across subjects.

Each network consists of sets of nodes (i.e., variables) listed in Table 1 and sets of edges describing the relationships between nodes. Blue and red edges portray positive and negative relationships, respectively. Importantly, each network model estimates the unique relationships among nodes while controlling for all other variables in the network. The main focus of the present study includes the average *within-subject* relationships (i.e., temporal and contemporaneous networks).

Centrality metrics [48] aim to quantify the role of individual nodes for the overall information flow in the networks. Strength centrality enhances the interpretation of network models through highlighting how strongly a node is directly connected to other nodes in the network. As a directed graph, the temporal network model enables estimation of the outstrength and instrength centrality, quantifying the sum of all outgoing and incoming absolute edge weights (i.e., excluding the autoregressive effect) from and to a node, respectively. Instrength thus reveals which nodes are more likely of being influenced by fluctuations in other nodes in the network at the previous day, while outstrength centrality quantifies the magnitude of a node in influencing other nodes in the network at the consecutive day. The undirected between-subject and contemporaneous networks provide estimations of strength centrality, computing the sum of all absolute edge weights connected to a node to quantify the overall weighted connectivity of the node in the network. All aforementioned strength centrality metrics reflect the average conditional associations between a node and the other nodes in a network. In the present study, we introduce a novel approach in *visualizing* centrality metrics using radar charts in order to enhance visual comparisons of centrality indices in a given network (i.e., outstrength versus instrength centrality in the temporal network) and across networks containing the same nodes. In line with the recommended reporting standards for network studies [49], we use raw centrality scores as opposed to standardized estimates, as the latter may inflate dissimilarity between centrality indices.

#### Sensitivity to demographic composition

The proportion of all demographic characteristics was investigated and compared to their known rate in the population. All characteristics not fully representative of the Norwegian adult population were adjusted in sensitivity analyses encompassing of a random selection of participants fully matching the population characteristics. The similarity and degree of replicability between the results from the main sample and the adjusted proportional subsample representative of the population were compared through correlating the respective matrices containing any estimated effects in the study, with its range reported at the beginning of the “Results” section.

#### Robustness and replicability of networks

Additional analyses were performed to assess the robustness and replicability of the network models. These analyses were conducted across four subdivisions of the dataset. First, all participants were randomly separated into two groups prior to re-estimation of the network models and assessment of the replicability of the findings across the two subsamples. Second, two additional subdivisions of the dataset were created, separating the data into an early subsection consisting of all participants using the first half of the time series and a second subsection encompassing of data of all participants using the latter half of the time series. Thus, the network models were further re-estimated to assess the replicability of the findings across the time-specific subsamples.

In each of the four aforementioned subsamples, three main analyses were conducted to assess the robustness of the findings, with each subsample compared to its respective counter-subsample as detailed above. First, following previous research [50], the global replicability and consistency of edges of each of three estimated networks (i.e., temporal, contemporaneous, and between-subjects network) was assessed through correlating the estimated edge weights in each subsample. Second, to assess the stability of centrality values, estimated centrality indices were compared through correlations across each respective pair of subsamples. Finally, the rate of consistency among the nodes with the highest centrality was assessed through comparing the total number of times the most central nodes identified by the main analyses were replicated across all four subsamples described above, used as a proxy to obtain estimations approximating the rank-order stability of the centrality indices.

In using such proposed assessments of robustness across subsamples, a previous study [50] found moderate replicability across subsample networks through correlations of.61 when comparing edge weights, toward which the present findings will be benchmarked against. The range of correlations derived from these robustness analyses is to be presented in the “Results” section labeled *network replicability*.

#### Network visualization

All networks have been visualized using the *qgraph* package in R [51]. The maximum edge weight across the three networks was set to correspond to the largest edge weight across the networks (i.e., *partial* *r*≈.4). Correspondingly, to filter out weaker from more notable effects, the minimum edge weight was set to one-tenth (i.e.,.04) of the maximum value. Note that the set minimum merely hides edges in the network figures for visualization and interpretation-enhancing purposes and does not remove them from the model. As common across dynamic network studies [44, 52], the temporal network generally exhibited smaller effects than the other two networks. Therefore, for visualization purposes, a cut value of.05 was set to more clearly separate the effects above and below this threshold. The arrangement of the nodes is based on the average layout of the three networks that have been established via the Fruchterman-Reingold algorithm [53]. The matrices containing all edge weights and the raw networks displaying all edges (i.e., including the weaker effects) can be found at the online repository of the Center for Open Science (https://osf.io/trf2y) and in Additional file 2: Figure S2-S4, respectively.

## Results

A total of 1706 participants enrolled in the study. The age of the participants ranged from 18 to 86 years (*M*_age_=37.30), with 1336 (78.54*%*) of the participants being female, 962 (56.89*%*) having a university degree, and 830 (49.43*%*) being married or in a civil partnership. A total of 1368 of the 1706 (80.19*%*) participants provided sufficient data to be included the study, with no pattern of difference identified between initiators and those with sufficient data. The percentage of individuals with preexisting mental health conditions in this sample was 16.62*%*, representative of the known rate of psychological disorders in the adult population of Norway, which is between 16.66 and 25.00*%* [54]. The sample was further geographically representative of Norway, with the quota of participants sampled from each region being proportional to region size. With the exception of sex and education (i.e., oversampling females and those with a university degree), the preponderance of demographic characteristics were representative of the Norwegian adult population. To fully match all demographic characteristics (i.e., including sex and education) to the known proportions in the population, sensitivity analyses were conducted on a randomly drawn set of 598 individuals fully matching the population parameters. These sensitivity analyses replicated the results from the main sample across all analyses below, with the correlation between the matrices containing the results of the representative sample and main sample ranging from.96 to.99.

### Time series analyses and time-specific patterns across the study period

Figure 1 provides a visualization of the time-specific patterns of depressive symptomatology and related constituents across the 40-day study period. Overall, mental health-promoting associations were identified during the holiday periods where pandemic protocols were lightened (i.e., days 6–12, 13–19, and 38 and onward), while detrimental associations were found during the period encompassing of uninterrupted viral mitigation protocols (i.e., days 20–37). Specifically, all unfavorable variables (e.g., loneliness, depressed mood, interpersonal conflict, helplessness) revealed linear decreases during holiday periods (*ps* <.001) while increasing during the continuous viral mitigation period (*ps* <.001). All favorable variables (e.g., relatedness) revealed linear increases during holiday periods (*ps* <.001), while decreasing during the uninterrupted viral mitigation period. The only notable exceptions from these patterns included (a) productivity (i.e., increasing during uninterrupted viral mitigation period, decreasing during holidays, *ps* <.001) and (b) lethargy, information access needs, sleep satisfaction, and rumination which did not reveal any significant fluctuations during the continuous viral mitigation period (*ps* >.05).

In-person (i.e., offline face-to-face) and digital social contact demonstrated opposite patterns, with in-person social contact decreasing during the continuous viral mitigation period and increasing during holidays, while digital social contact decreased during holidays and increased during the continuous viral mitigation period (*ps* <.001).

### Patterns of interplay between depressive symptoms and related components

The *within-person* patterns of interplay obtained in the temporal and contemporaneous network models (Fig. 2) provide insight concerning the potential processes involved in the maintenance and amplification of depressive symptomatology.

**Fig. 3:**
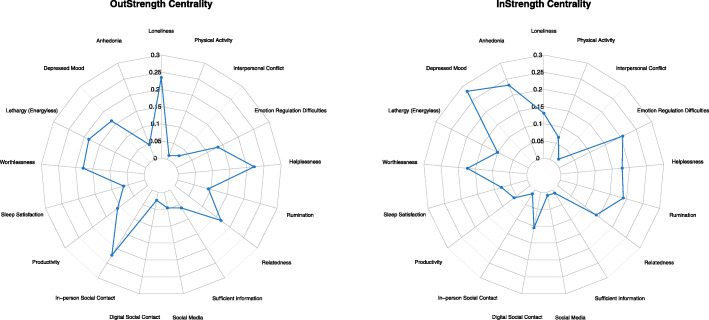
Radar chart depicting the OutStrength (i.e., sum of all outgoing absolute edge weights from a node) and InStrength centrality (i.e., sum of all incoming absolute edge weights to a node) of the variables in the temporal network model. The across-day directed involvement of a node is revealed through the extent of which a node influences other nodes (i.e., OutStrength) at the consecutive day or is influenced by other nodes in the network at the previous day (i.e., InStrength)

Figure 2 (top panel) displays the temporal network revealing the average *within-person* connections between nodes *from one day to the next*, with the radar plots in Fig. 3 depicting each variable’s outstrength and instrength centrality. The radar plots depicting outstrength and instrength displayed distinctive patterns, indicating differences in the extent to which nodes were associated with having outward influencing roles versus susceptibility of being influenced on an across-day basis. Loneliness, helplessness, and in-person social contact had the greatest outstrength centrality. Depressed mood, anhedonia, and emotion regulation difficulties had the greatest instrength centrality. Concerning node connections, specific across-day patterns unfolded between lethargy and anhedonia, with greater within-person levels of lethargy temporally predicting increases in within-person levels of anhedonia at the consecutive day and anhedonia further reinforcing itself across days in a vicious self-loop. This pattern of interwovenness also involved an autoregressive carry-over effect in lethargy, in which low energy levels carried over across days. The across-day interplay among depressive symptomatology was coupled and separated, with lethargy and anhedonia representing one pair, while depressed mood and worthlessness represented the other. Helplessness was among the nodes revealing the highest outstrength centrality across days, with higher within-person levels of helplessness being involved in the amplification of other detrimental mechanistic processes (i.e., increases in rumination and emotion regulation difficulties) in addition to key symptoms of depression (i.e., increases in depressed mood and worthlessness), all further involved in detrimental self-loops across days. A vicious cycle was identified between helplessness and emotional regulation difficulties, with higher within-person levels of each predicting greater increases in the other at the consecutive day. Examples of across-day patterns with smaller magnitude included the directed effects from relatedness to loneliness (i.e., higher within-person levels of relatedness at the previous day predicted less loneliness at the consecutive day), greater helplessness predicting more worthlessness the next day, and more emotion regulation difficulties and loneliness predicting higher depressed mood at the following day. Additionally, although having smaller magnitude in its outgoing effects, in-person social contact demonstrated widely distributed across-day influence on the other nodes in the network, as reflected by its position among the nodes with greatest outstrength (Fig. 3). This widely distributed outgoing connectivity is visible in the raw network containing all effects found in Additional file 2: Figure S2.

**Fig. 4:**
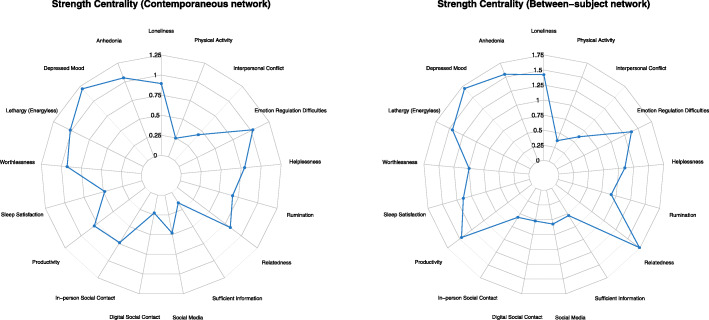
Radar chart revealing the strength centrality (i.e., the sum of all absolute edge weights connected to a node), quantifying the overall weighted connectivity of the node in the contemporaneous (left) and between-subject (right) network models

Inspecting the contemporaneous network (Fig. 2, middle panel) provides indications of average *within-person* relationships among the investigated nodes occurring *within the same window of measurement*, which in the present study reflects a within-day time window. All abovementioned relationships between depressive symptoms and related constituents were present within the same window of measurement. In contrast to the across-day patterns including separate clusters of interaction among depressive symptoms, all depressive symptoms were related with one another in the contemporaneous network. Notable unique patterns of interconnection were found within a daily window of measurement, with within-person sleep satisfaction inversely related to lethargy, within-person increases in loneliness associated with higher within-person levels of anhedonia and lower relatedness, and greater emotion regulation difficulties being associated with more interpersonal conflict and worthlessness. Additionally, productivity portrayed negative within-day relationships with both anhedonia and lethargy. Importantly, the relationship between rumination and key depressive symptoms (i.e., worthlessness and depressed mood) predominantly occurred within the same window of measurement (i.e., within a day), revealing weak effects across days. Among the contextual variables prominent during the pandemic, in-person social contact and relatedness were further strongly interwoven in the same time window. The most central (i.e., strength centrality; Fig. 4, left panel) nodes in contemporaneous network were depressed mood, anhedonia, and emotional regulation difficulties, outlining the nodes with the strongest overall connectivity within a day among the nodes in the network.

### Risk factors associated with depressive symptoms

The between-subjects network (Fig. 2, bottom panel) is suitable in the identification of risk factors across subjects in a population. Several distinctive associations were derived from this network, predominantly involving the contextual variables of the study. Particularly, the relationship between information access needs and sleep quality was highlighted, revealing that people who on average feel well-informed about the pandemic also report greater sleep satisfaction compared to other adults in the population. Higher relatedness was associated with greater productivity across subjects. Similarly, there was a negative association between productivity levels and perceptions of worthlessness, and a positive association between productivity levels and sleep satisfaction. Overall, the nodes with the highest strength centrality (Fig. 4, right panel) in the between-subject networks were relatedness, depressed mood, and anhedonia.

### Network replicability

The estimated network models and their corresponding computed parameters were yielded as robust, replicating the main findings. Specifically, the correlation between edge weights comparing the two random subsamples of participants was *r*=.93 for the temporal network, *r*=.99 for the contemporaneous network, and *r*=.97 for the between-subjects network. Correspondingly, the correlation between edge weights comparing the first half of the time series compared with the latter half was *r*=.92 for the temporal network, *r*=.99 for the contemporaneous network, and *r*=.99 for the between-subjects network. Centrality estimates were further robust across both aforementioned pairs of subsamples, with correlations ranging from *r* =.89–.96 (i.e., instrength) to *r* =.80–.88 (i.e., outstrength) for the temporal network, stable at *r*=.99 for the contemporaneous network, and ranging from *r* =.88 to.97 for the between-subject network.

Finally, the edges revealing the highest centrality were consistent across all subsamples (cf. Additional file 3: Table S1), with 96.67*%* of the edges with the highest centrality re-obtained in the subsample analyses across all networks.

## Discussion

As discussed by multiple scholars [8, 11–14], a study of within-person relationships is required to understand the mechanisms of change in human behavior and psychopathological research. The disentanglement of such within- and between-person relationships are imperative, as conclusions from one level do not necessarily generalize to the other, where in extreme cases, these relationships can convey opposite patterns [8, 11, 12, 14]. Consequently, understanding the maintaining components involved in depressive states necessitates the study of within-person relationships.

### Maintaining mechanisms of depressive symptomatology

The main purpose of the present study was to examine the *within-person* relationships present in the temporal and contemporaneous models of depressive symptoms and its constituents. As these networks model average within-person connections between nodes, they provide insight into potential mechanisms of change involved in the amplification and impediment of depressive symptomatology, providing directions toward further study and identification of targets for interventions aimed at alleviating these detrimental mental health problems.

Although all depressive symptoms were well-connected on a between-subject level and further interacted within the same window of measurement, the findings of present study indicate that interactions between depressive symptoms to a greater extent are separated and uniquely coupled across days. Specific across-day connections were identified between anhedonia and the somatic symptom lethargy, while two cognitive-affective symptoms, perceptions of worthlessness and depressed mood, were more strongly interconnected on an across-day basis. Additionally, the relationship between these symptoms were directed, revealing the predominant temporal influence of lethargy on anhedonia, and worthlessness on depressed mood. These findings have implications for efforts aimed at impeding escalations of depressive states, suggesting that lethargy and worthlessness have a greater likelihood of contributing as catalysts in the escalation of deleterious depressive states from one day to the next. As a key feature putting individuals at risk of developing depressive syndrome involves the prolonged constellation and experience of multiple symptoms [40], insight into the specific symptoms that more likely yield carry-over effects across time is of importance from an epidemiological and clinical perspective in more successfully preventing the development of a depressive state. The present study identifies that the two most prominent depressive symptoms that may be involved in such detrimental carry-over effects in the non-clinical population are worthlessness and lethargy. This finding is consistent with cross-sectional network studies identifying worthlessness and lethargy as central nodes in depressive states [16, 17], with the present study advancing insight concerning the directed temporal involvement and coupled interaction between these symptoms.

This investigation further extended the applications of network theory through the introduction of relevant psychopathological mechanisms and contextual factors in the networks, yielding novel insights concerning the specific patterns that these processes exhibit in their interactions with depressive symptomatology. Loneliness, helplessness, and in-person social contact had the greatest outward temporal influence (i.e., outstrength centrality) on the other variables in the network on an across-day basis. Studies during the present pandemic have found undirected associations between loneliness and depressive symptomatology in the general population [55, 56]. The present longitudinal study advances the literature by identifying the direction of this association, further identifying that loneliness interacts with depression through its directed association with the depressed mood component of depression, carrying over across days.

The main psychopathological mechanism temporally associated with the maintenance and amplification of depressive dynamics on an across-day basis was helplessness. Accordingly, when an individual reported being more helpless than their own average at a given day, they reported within-person increases in depressed mood, rumination, and worthlessness at the consecutive day. This finding provides support for helplessness as an important mechanistic variable in the maintenance and change of depressive symptoms in the general population. This is consistent with the learned helplessness theory of depression [26], postulating that when an individual comes to believe that their efforts to modify their circumstances are ineffective, developed perceptions of helplessness may incite depressive symptomatology. The finding is further consonant with a central meta-theory of psychopathology proposed by Jerome Frank, suggesting that demoralization (i.e., experienced helplessness or inability to cope) is a key aggravator of psychiatric symptomatology [57]. As perceptions of helplessness are theorized by several scholars to be the main reason for individuals seeking psychiatric treatment [57, 58], directing efforts toward reducing helplessness may be warranted. The present study provides preliminary indications that such efforts may have the ability to impede deleterious depressive states, although such assertions warrant further investigation using controlled designs.

Aside from being uniquely associated with within-person increases in key depressive symptoms and rumination at the next day, helplessness was further engaged in a vicious cycle with emotional regulation difficulties across days, with emotion regulation problems also associated with heightening of depressed mood from one day to the next within individuals. Combined with the finding that emotion regulation difficulties were the most central psychopathological process in the contemporaneous network, revealing strong interactions with depressive symptoms within a day, this finding suggests it may be important to devote simultaneous attention toward the detrimental role that emotional regulation difficulties may play in depressive mental health states. Notably, this study provides indications that the interaction between depressive symptoms and emotional regulation difficulties may predominantly operate on a faster time scale than helplessness with depressive symptoms. This finding is meaningful, given that emotional regulation problems likely are more situationally contingent and probable of occurring within a more encapsulated time period. Consequently, these findings distinguish between the proximal role that emotion regulation difficulties play in its interaction with depressive symptoms, while identifying helplessness as having a more prominent role in terms of prolonged depressive symptom experience. More granular approaches are called for in future studies to refine the understanding of the possible directed role that emotion regulation difficulties may play within a day.

Among the aforementioned psychopathological mechanisms, rumination was peripheral and did not have any notable interaction with depressive symptomatology on an across-day basis. This finding is consistent with a previous study [59] identifying rumination to be on the receiving end of predictive temporal relationships in a network of mechanistic variables, in addition to another study not finding any temporal relationship between rumination and depressive symptoms [60]. In the present study, the only notable connection with rumination included a directed effect from helplessness predicting rumination at the consecutive day. This finding suggests that helplessness may play a more prominent role in the maintenance and across-day constellation of depressive symptomatology in the non-clinical population, consistent with the goal progress theory of rumination proposing rumination to be a response to failure in achieving a certain task rather than an outgoing mechanistic process [61]. Consistent with existing studies [16], rumination revealed undirected associations to some symptoms of depression (e.g., weaker associations with worthlessness) on both a between-subject level and within a day. However, the present findings in combination with findings from directed network studies investigating within-day relationships involving depression and rumination [59, 60] provide indications that these associations may to a greater extent be indicative of rumination being an influenced node rather than the influencing node, with implications for interventive efforts aimed at alleviation of depressive symptoms. This finding is further partially consistent with metacognitive perspectives on depression [25], postulating rumination to be a process *ensuing* depressive symptoms as a reactional attempt to understand the reason for their presence and in attempts of identifying solutions to the problem. However, the present study does find indications of rumination subsequently influencing depressive symptoms in turn, which is also postulated by the theory. Still, given the multimodal complexity of rumination [62, 63], the literature will benefit from further temporal examinations of depressive symptoms simultaneously investigating rumination along with other psychopathological mechanisms of relevance, to better understand its specific as well as comparative interaction with depressive components.

The findings of the present study further shed some light on the interactions between depressive symptomatology and mechanistic processes that operate on a faster time scale than an across-day basis. In the present study, this reflects the identified interactions in the contemporaneous network, which cautiously provide indications of associations among nodes that may occur within person during a given day. Meaningful connections emerged between lethargy within individuals in its association with reduced sleep satisfaction within the same time window, while being more productive than usual was associated with lower anhedonia and lethargy. Loneliness was a central node with important connections to depressive symptoms and contextual variables across all three networks. On a within-person level, loneliness displayed its largest connectivity within a day, with the findings indicating that while individuals felt greater loneliness than their own average, this was associated with greater within-person intensity of depressed mood and anhedonia. Consistent with a study by Fried and colleagues [44] on the student population, the present study found higher within-person levels of loneliness to be associated with reduced relatedness and in-person contact. The present study supports and adds to these findings by extending the time period of investigation to later stages of the pandemic and a broader demographic composition of participants, in addition to identifying detrimental associations between loneliness and depressed mood.

Notably, on the within-person level, the three psychopathological processes (i.e., helplessness, rumination, and emotion regulation difficulties) only exhibited interactions with the depressed mood and worthlessness component of depression, being unrelated to lethargy and anhedonia. These findings highlight the connection between these aforementioned cognitive-affective mechanisms with particular depressive components, providing important insights on the patterns of interaction between depressive symptoms and mechanistic processes. Simultaneously, they also leave important gaps in the literature concerning the identification of pathological processes more closely intertwined with lethargy and anhedonia on the within-person level.

### Risk factors associated with depressive symptoms across subjects

Across subjects, in-person social contact was revealed as the type of social interaction with the strongest association with relatedness, with those who reported being more frequently engaged with such face-to-face contact compared to their peers also reporting greater relatedness. Moreover, individuals who on average felt more connected to their peers during the pandemic reported greater levels of productivity, further mirrored by within-person relationships to outline several beneficial associations of relatedness. However, although relatedness was connected to anhedonia on a between-subject level, this connection was not present in any of the within-person networks (i.e., temporal and contemporaneous network). This demonstrates the importance of separating between- and within-person effects [8, 11, 12, 14], with this finding implying that it is unlikely that relatedness is directly associated with anhedonia. Rather, as also revealed by the within-person networks, relatedness is more indirectly connected to depressive symptoms through its association with loneliness.

Between-person associations were further identified between information access and sleep, with those who on average reported sufficient access to information about the pandemic situation reporting greater sleep satisfaction compared to their peers. Still, no within-person relationships emerged for this association. Moreover, no social contact component other than in-person social contact revealed notable beneficial associations across any of the investigated networks, with other social contact components additionally portraying detrimental associations to depressive states. Specifically, consistent with previous findings [31, 64], individuals who compared to their peers who were more engaged in passive social media use had a greater risk of being associated with higher levels of anhedonia, in addition to lower productivity. Yet, again, however, no meaningful within-person detrimental association emerged between social media use and anhedonia, suggesting the limited likelihood of this factor being associated with within-person fluctuations in depressive states when controlling for all other variables in the network. Additionally, no beneficial within-person associations were identified with digital social contact. Taken together, these findings highlight solely in-person social contact as having a potentially important role on a within-person basis through this variable association with loneliness and relatedness. As loneliness is an important problem in itself [36] and further was found to be connected to depressed mood across days on the within-person level in this study, this finding implies that attempts to find an optimal balance between strength of viral mitigation protocols and appropriate levels of in-person social contact, the latter of which the present findings reveal to be hard to substitute by other social contact types, may be of utility in combating the concurrently ubiquitous presence of loneliness. Clever behavioral interventions at the population level, including the use of social bubbles, may serve as utile strategies that can simultaneously reap the psychological benefits of reduced loneliness while maintaining control over viral spread [65]. As for depressive symptoms, however, the present study does not identify any direct within-person relationship between social contact and depressive symptomatology, suggesting that efforts toward alleviation of depressive symptoms may be more fruitful when aimed at other identified mechanistic and contextual variables.

### Other notable findings

The social contact components were negatively associated in the contemporaneous network, reflecting that while an individual is engaged in a greater extent of in-person social contact than their own average, they are less involved in digital social contact within the same window of time. This stands in informative contrast with the positive associations between these components in the between-subject network, which highlights that people who on average are more engaged with in-person social contact compared to their peers likely also are people who to a greater extent are engaged in both social media use and digital social contact. In other words, social individuals are sociable, likely to report higher levels of engagement compared to their peers among a wide range of social activities (i.e., between-subject network), but being engaged with one social activity in a given time window reduces the opportunities of being engaged with another social activity within the same time window (i.e., contemporaneous network). This contrasting finding between the two networks highlights the importance and utility of disentangling between within-person and between-person relationships. This is further emphasized through the positive connection identified between emotion regulation difficulties and worthlessness on a within-person level, while this relation was absent across individuals. In other words, while individuals experienced greater emotional regulation difficulties than their own average, this was associated with increased feelings of worthlessness during that day (i.e., a within-person effect). However, individuals who have greater emotion regulation difficulties compared to their peers are not likely to be individuals who feel worthlessness. Within-person and between-person relationships are not necessarily coherent, and the inappropriate generalizations from the between- to the within-level has been referred to as ecological fallacy [8, 66]. In its investigation of within-person relationships among multiple theorized detrimental processes, the present study fills the gaps [7, 17] in progressing the understanding of psychopathological mechanisms connected to depressive symptomatology in the general population.

Moreover, physical activity and digital social contact were consistently among the least central and influential node across all networks, outlining their limited relevance and involvement in depressive states when controlling for all other nodes in the network during the present pandemic context. Specifically, as no particularly notable within-person relationship was present between these variables and depressive symptoms, the present findings suggest that future efforts toward identification of variables that may impede deleterious symptoms within subjects best are aimed at other central components of symptom maintenance, such as helplessness and emotion regulation skills building. The findings of the present study thus imply that promising interventive targets warranting investigation in future controlled studies may include testing whether and how techniques such as cognitive restructuring and behavioral activation may temporally interact and impact perceptions of helplessness and lethargy, respectively.

Finally, this study introduces the usage and utility of radar plots in visualizing key information about network centrality metrics, with the results of the temporal network model outlining the comparative extent of involvement of a given node as an outgoing node at an across-day basis versus as a node more strongly tied to being influenced from other nodes at the previous day. Both loneliness, helplessness, and lethargy had greater strength as outgoing nodes in contrast to being influenced. As relationships in temporal networks are indicative of Granger causal effects, these findings preliminary indicate the greater likelihood that helplessness, loneliness, and lethargy may play in serving as engines in the network, to a greater extent being associated with activation of other nodes. However, as Granger causal effects do not necessarily equate true causal processes and only satisfy its temporal criterion, these findings warrant further investigation in future studies. Other drastic differences were found for in-person social contact in terms of its relative position as an influential node versus being influenced, a finding which is meaningful in the present pandemic setting.

Both anhedonia and depressed mood were more likely to be impacted by other nodes at the previous day than having across-day carry-over effects. Across three of four centrality estimations (i.e., with the exception of outstrength centrality), depressed mood and anhedonia were the most central nodes in the networks, which provides support for their position as the key identifiers of depression [40]. Importantly, however, these findings illuminate their more limited outgoing involvement in depressive states, highlighting lethargy and worthlessness to have stronger outgoing impact on other symptoms.

### Strengths and limitations

The present paper consists of several limitations. First, the conclusions of this paper have to be interpreted in light of the underlying assumptions of the statistical model. More specifically, we interpreted a lack of relationships in the temporal network as indicative of potentially faster interactions between depressive symptoms and related components [24]. This interpretation assumes that meaningful interactions can in principle be captured using linear lag-1 models. An alternative explanation for the lack of detected temporal relationships is that these could be nonlinear or time-varying [67–70], which calls for further investigations using other modeling approaches. Furthermore, although the study investigated some of the most central theorized mechanisms in the psychopathological literature, the edge weights were generally smaller in the temporal network than the other networks, as commonly the case in multi-level network analytic studies [44, 52]. This further highlights the necessity of advancing current and building novel theories through formalization and incorporation of the time-scales which phenomena may operate on [70, 71]. Finally, the modeled relationships in the present paper are on the average within-person level, calling for idiographic efforts [72] in inspecting how closely such within-person aggregations correspond to the level of the individual.

This study consists of several strengths, including that it was pre-registered with a clear rationale preceding the selection of variables. Additional strengths include the use of validated measures, its focused time window of measurement corresponding to the DSM-V depressive symptom endorsement assessment, longitudinal design, broad demographic composition of participants, and conducted sensitivity analyses on a fully representative sample replicating the main findings. Moreover, the robustness and replicability of the network models and their corresponding estimated parameters were assessed across four additional subsamples, revealing high robustness of the results. Importantly, the investigation of psychopathological mechanisms in a non-clinical population provides insight into the processes that may be involved in the formation and maintenance of detrimental depressive states which may turn to more enduring problems. A major strength of the present study includes the focus on within-person rather than between-person relationships. This is an asset because theories in psychopathology concern how within-person change in a mechanism variable relates to within-person change in symptoms. Important differences were identified between these two divergent types of relationships, providing clearer directions concerning promising targets for intervention that should be investigated in future studies. The present study is among the largest intensive longitudinal investigations of psychopathology in the adult population, contributing to the stability of its results. Further efforts to assess the replicability of the presented findings in independent samples and in the clinical population would benefit the literature. Finally, the use of longitudinal data and multi-level approach is powerful and overcomes many of the short-comings experienced in dynamic modeling.

### Conclusions

In identifying psychopathological mechanisms and central symptoms involved in the maintenance of depressive states, investigations of within rather than between-person relationships are needed. This intensive longitudinal study identified helplessness as the main mechanism interwoven with depressive symptomatology on an across-day basis, while emotion regulation difficulties had more proximal associations with depressive symptoms. While depressed mood and anhedonia were identified as symptoms most susceptible toward being influenced by other nodes in the network, the present study identified that the two most prominent symptoms displaying outward temporal influence were worthlessness and lethargy. These symptoms had greater within-person carry-over effects across days, putting individuals at greater risk of prolonged depressive state experiences. This suggests that not all symptoms of depression should be viewed as equal in their role in maintaining this deleterious mental health state. Finally, rumination was to a greater extent susceptible to being influenced rather than temporally influencing other components involved in depressive states. These findings outline several associations between symptoms and mechanisms that are important to investigate further toward advancing the etiological understanding of depression.

## Supplementary Information

**Additional file 1** **Figure S1**: Cumulative length of time-series per person across the study participants.

**Additional file 2** **Figure S2-S4**: Figure S2 - Supplementary temporal network with all effects. Figure S3 - Supplementary contemporaneous network with all effects. Figure S4 - Supplementary between-subjects network with all effects.

**Additional file 3** **Table S1**: Consistency amongst highly central nodes across replicability analyses.

## Data Availability

The materials necessary to regenerate the estimated models of the present study may be found at the online repository of the Center for Open Science (https://osf.io/trf2y). As our received ethical approval from the Norwegian Centre for Research Data (NSD) precludes submission of raw data to public repositories, the matrices underlying the model estimation are provided. Access to the data can be granted from the principal investigators Omid V. Ebrahimi and Sverre Urnes Johnson following ethical approval of a suggested project plan for the use of data granted by NSD and REK. All code for the present study is uploaded at the online repository of the Center for Open Science (https://osf.io/m2zhu/). We also provide a step-by-step guide for conducting radar plot visualizations of centrality metrics, readily available in the code.

## Abbreviations

mlVAR: Multi-level vector autoregressive (VAR) model
DSM-V: Diagnostic and Statistical Manual of Mental Disorders, fifth edition
