# Supplementary figures and images for: Within- and across-day patterns of interplay between depressive symptoms and related psychopathological processes: a dynamic network approach during the COVID-19 pandemic
Source: BMC Med. 2021 Nov 30;19:317. doi: 10.1186/s12916-021-02179-y (PMC8629696; doi:10.1186/s12916-021-02179-y)

Figure S1. Cumulative length of time-series per person across the study participants

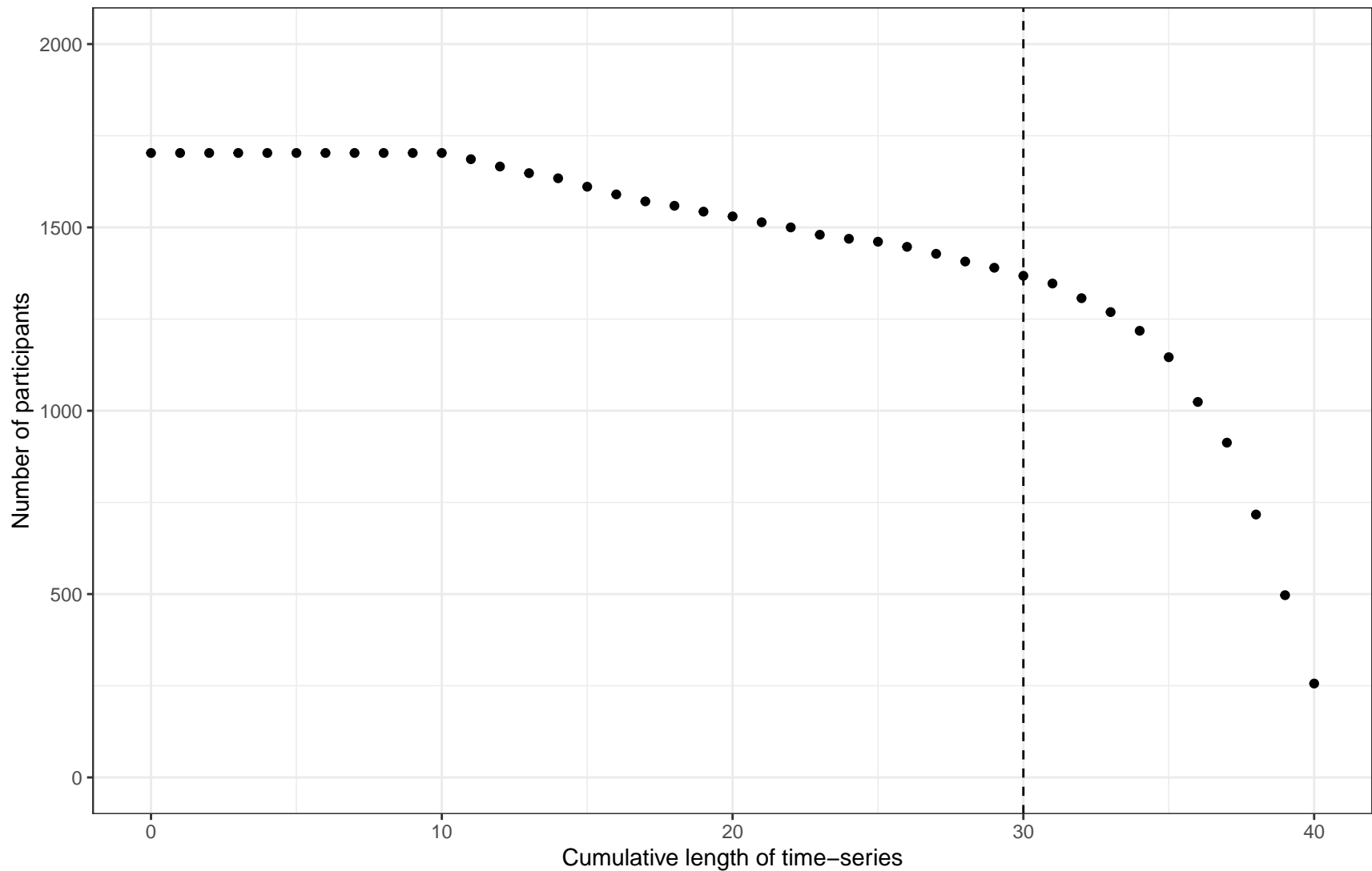

Supplement: Supplementary file 1 — Additional file 1 Figure S1: Cumulative length of time-series per person across the study participants. [file 12916_2021_2179_MOESM1_ESM.pdf]
