# Supplementary material for: Within- and across-day patterns of interplay between depressive symptoms and related psychopathological processes: a dynamic network approach during the COVID-19 pandemic
Source: BMC Med. 2021 Nov 30;19:317. doi: 10.1186/s12916-021-02179-y (PMC8629696; doi:10.1186/s12916-021-02179-y)

Figure S2. Supplementary temporal network with all effects

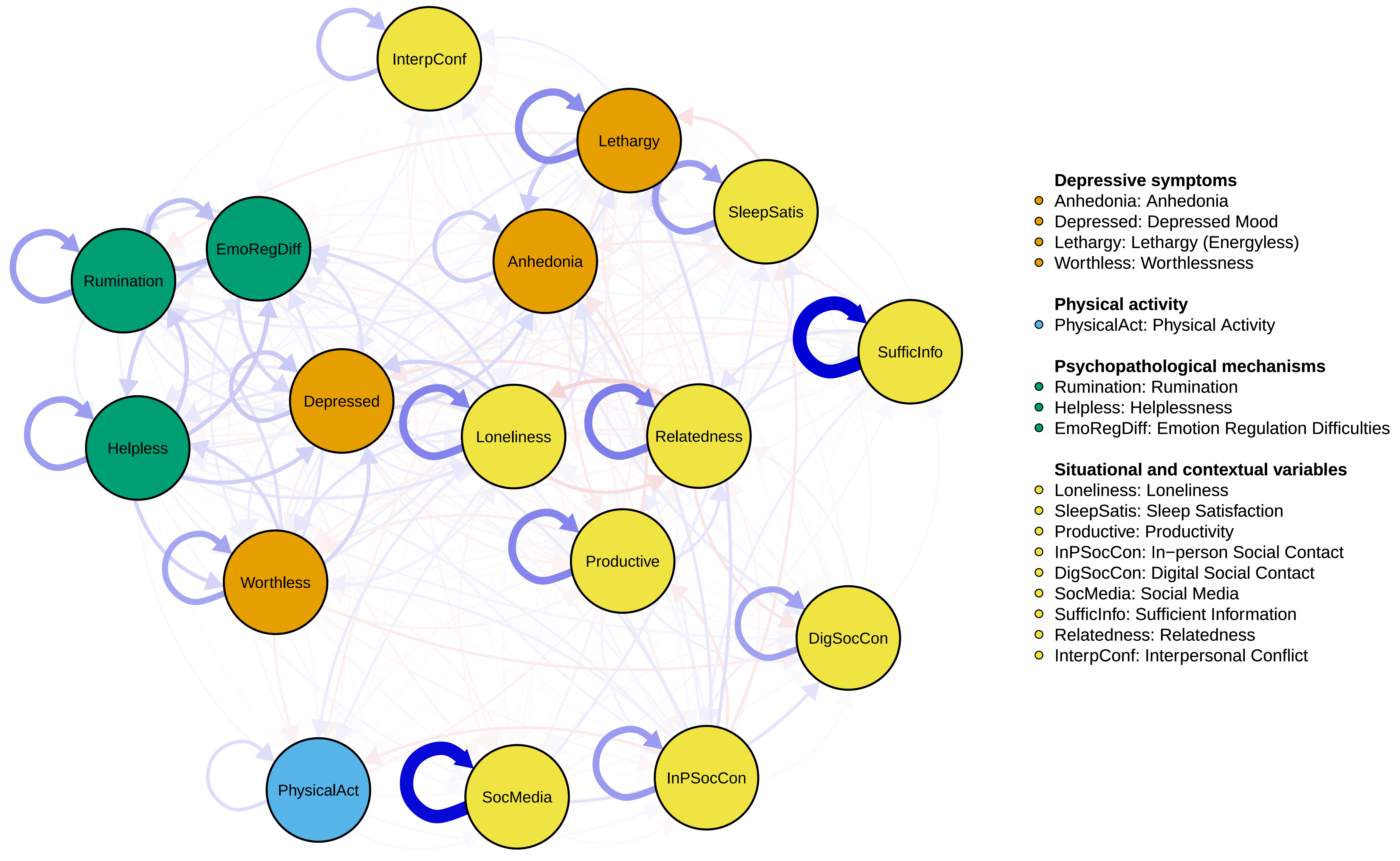

Figure S3. Supplementary contemporaneous network with all effects

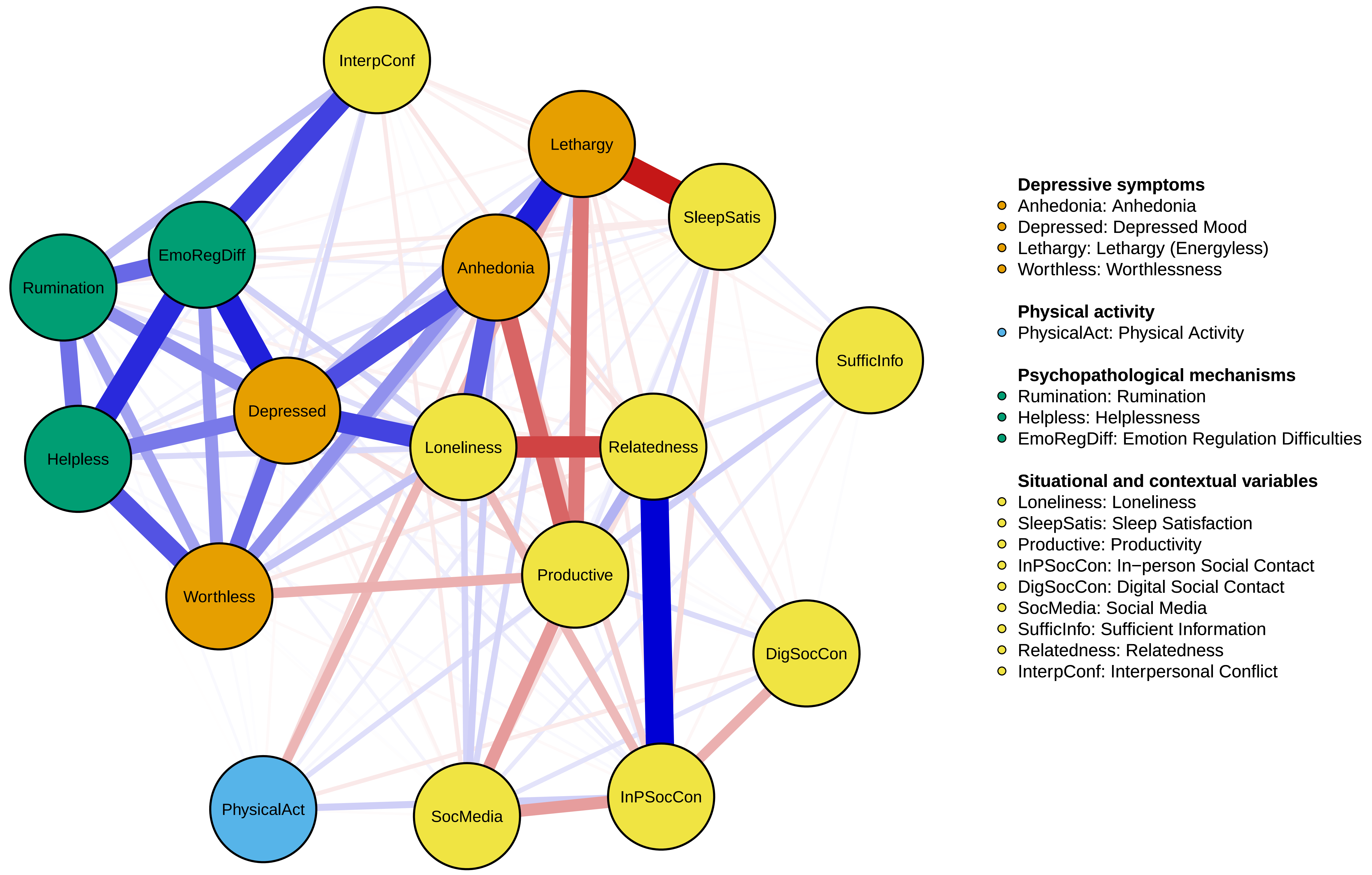

Figure S4. Supplementary between−subjects network with all effects

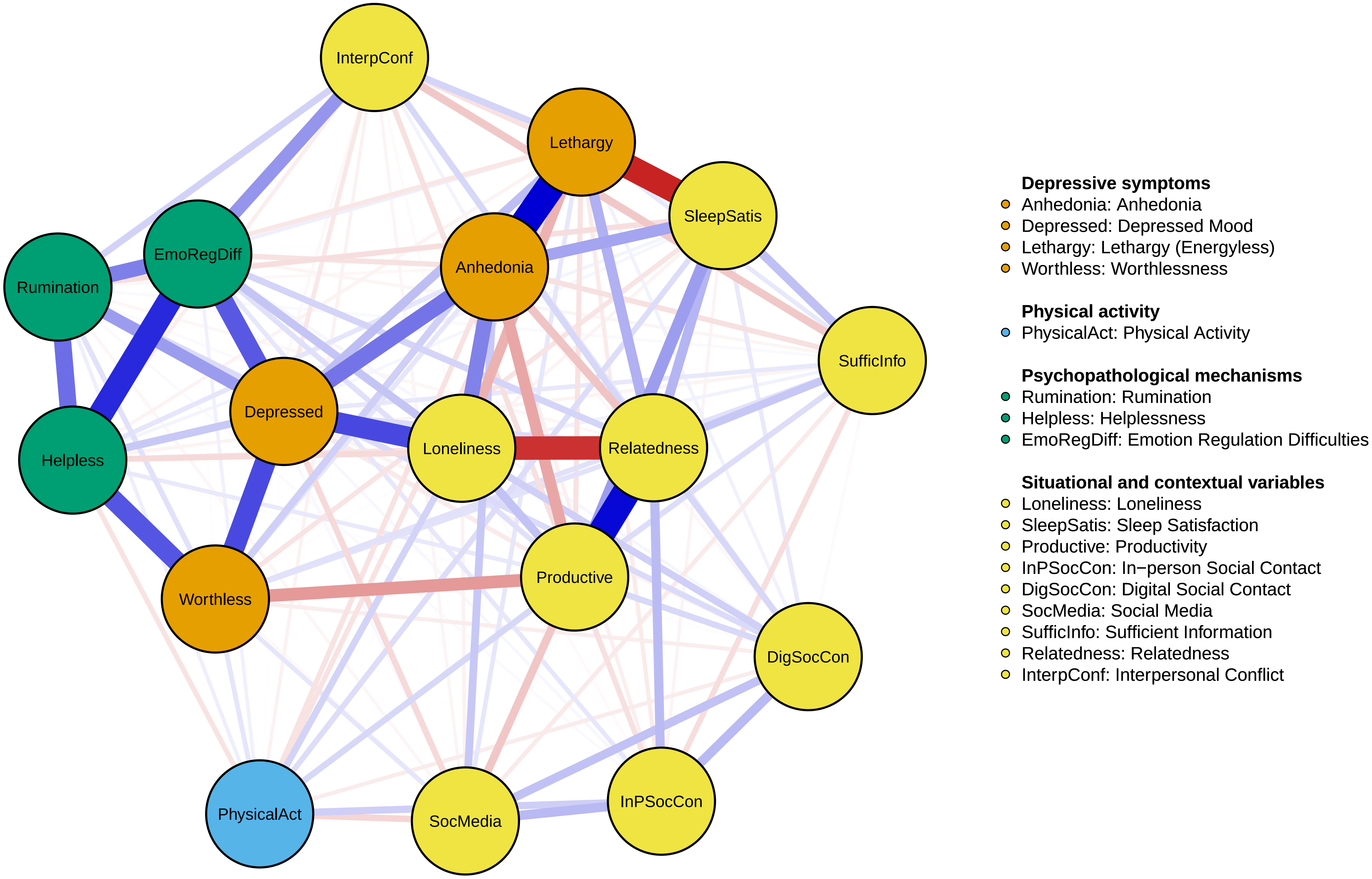

Supplement: Supplementary file 2 — Additional file 2 Figure S2-S4: Figure S2 - Supplementary temporal network with all effects. Figure S3 - Supplementary contemporaneous network with all effects. Figure S4 - Supplementary between-subjects network with all effects. [file 12916_2021_2179_MOESM2_ESM.pdf]
